# Breast cancer patients’ clinical outcome measures are associated with Src kinase family member expression

**DOI:** 10.1038/sj.bjc.6605829

**Published:** 2010-08-17

**Authors:** B Elsberger, R Fullerton, S Zino, F Jordan, T J Mitchell, V G Brunton, E A Mallon, P G Shiels, J Edwards

**Affiliations:** 1 Western Infirmary Glasgow, Section of Surgery, Division of Cancer Sciences and Molecular Pathology, Faculty of Medicine, Level 2, McGregor Building, Dumbarton Road, Glasgow G11 6NT, UK; 2 Western Infirmary Glasgow, Division of Developmental Medicine Reproductive and Maternal Medicine, Faculty of Medicine, Level 3, McGregor Building, Dumbarton Road, Glasgow G11 6NT, UK; 3 Edinburgh Cancer Research Centre, University of Edinburgh, Crewe Road, Edinburgh EH4 2XR, UK; 4 Western Infirmary Glasgow, Department of Pathology, Dumbarton Road, Glasgow G11 6NT, UK

**Keywords:** Src kinase family members, breast cancer, quantitative real-time PCR, immunohistochemistry, disease-specific survival

## Abstract

**Background:**

This study determined mRNA expression levels for Src kinase family (SFK) members in breast tissue specimens and assessed protein expression levels of prominent SFK members in invasive breast cancer to establish associations with clinical outcome. Ki67 was investigated to determine association between SFK members and proliferation.

**Methods:**

The mRNA expression levels were assessed for eight SFK members by quantitative real-time PCR. Immunohistochemistry was performed for c-Src, Lyn, Lck and Ki67.

**Results:**

mRNA expression was quantified in all tissue samples. *SRC* and *LYN* were the most highly expressed in malignant tissue. *LCK* was more highly expressed in oestrogen receptor (ER)-negative, compared with ER-positive tumours. High cytoplasmic Src kinase protein expression was significantly associated with decreased disease-specific survival. Lyn was not associated with survival at any cellular location. High membrane Lck expression was significantly associated with improved survival. Ki67 expression correlated with tumour grade and nuclear c-Src, but was not associated with survival.

**Conclusions:**

All eight SFK members were expressed in different breast tissues. Src kinase was highest expressed in breast cancer and had a negative impact on disease-specific survival. Membrane expression of Lck was associated with improved clinical outcome. High expression of Src kinase correlated with high proliferation.

In 1911 Peyton Rous discovered v-Src, an avian retrovirus, causing transmissible sarcoma in chicken. This was followed by the discovery of c-Src, the human cellular counterpart of v-Src (Stehelin *et al*, 1976). It has now been established, that c-Src is part of a family of non-receptor tyrosine kinases containing eight family members expressed in mammalian cells that are involved with cancer progression and invasion (Brown and Cooper, 1996; Manning *et al*, 2002). c-Src, Fyn and Yes are widely expressed, whereas Lck, Hck, Fgr, Blk and Lyn are more selectively expressed in specific tissues (Irby and Yeatman, 2000; Palacios and Weiss, 2004). All Src family kinase (SFK) members have a similar structure: a C-terminal tail, four conserved Src homology domains and a unique amino-terminal domain that varies between the family members (Martin, 2001; Engen *et al*, 2008).

Src kinase has been investigated for a long time in a variety of solid tumours. Data from human cancer tissues have further defined the role of Src in tumour development in a more clinically relevant setting. Elevated levels of Src or SFKs have been detected in a range of human solid tumours, including glioblastoma (Kleber *et al*, 2008), cancers of the prostate (Tatarov *et al*, 2009), breast (Verbeek *et al*, 1996), pancreas (Fu *et al*, 2006), colon (Bolen *et al*, 1987) and lung (Masaki *et al*, 2003). *In vitro* evidence for a role for c-Src in breast cancer is convincing, but currently hardly supported by translational clinical studies. In breast cancer, Src activity and distribution might impact on resistance to endocrine therapy in patients with oestrogen receptor/progesterone receptor (ER/PgR)-positive disease. Elevated c-Src activity promotes cellular invasion and motility in tamoxifen-resistant breast cancer cells (Hiscox *et al*, 2006) and provides a link between the HER family and steroid receptors (Yeatman, 2004; Likhite *et al*, 2006). Campbell *et al* (2008) illustrated that activated Src localised to the nucleus was significantly associated with improved overall survival and a lower recurrence rate during tamoxifen treatment of ER/PgR-positive tumours.

Other Src family members have also been linked with breast cancer. Again, there is little published evidence on the role of other Src family members in clinical breast cancer specimen. It is well established that Lyn has an important role in leukaemia. This has been suggested by several studies (Roginskaya *et al*, 1999; Wilson *et al*, 2002; Warmuth *et al*, 2003). Lyn is also involved in the development of certain solid tumours. Colon carcinoma cells use Lyn in the activation of the Akt (anti-apoptotic) pathway, and chemoresistant colonic cancer cells displayed elevated Lyn kinase activity (Bates *et al*, 2001). Inhibition of Lyn in prostate cancer cell lines resulted in reduced proliferation *in vitro* and in prostatic cancer xenograft models (Goldenberg-Furmanov *et al*, 2004). A recently published study shows that Lyn was associated with shorter overall survival, and that RNAi knockdown of Lyn in breast cancer cell lines inhibited cell migration and invasion, but not proliferation (Choi *et al*, 2010). Microarray studies have demonstrated that Lyn is induced in models of endocrine resistance (Gee *et al*, 2006) and Lck is implicated in hypoxia-induced breast cancer progression (Chakraborty *et al*, 2006).

The aim of the present study was therefore to establish mRNA expression levels for SFK members in human breast tissue and, subsequently, to assess protein expression of the most abundantly expressed SFK members, in a larger cohort of invasive breast cancer patients, to determine whether these are linked to clinical outcome measures.

## Materials and methods

### Patients

The study was granted approval by the local ethics committee for both the cohort used to determine mRNA expression (reverse transcriptase PCR (RT–PCR) cohort) and the cohort used to determine protein expression (immunohistochemistry (IHC) cohort).

The RT–PCR cohort contained 139 patients and was subdivided into Patient group 1 (M), consisting of malignant tissue samples taken from 81 breast cancer patients at the time of primary tumour resection. All patients were diagnosed with invasive breast carcinoma between 1987 and 2005 in the Greater Glasgow area. Patient group 2 (NM) included non-malignant tissue samples from 48 breast cancer patients taken from disease-free areas of mastectomy resection specimens. Patient group 3 (N) comprised of 10 normal breast tissue specimens obtained from reduction mammoplasties. The ER status was determined in a routine diagnostic laboratory.

The IHC cohort is completely distinct from the PCR cohort with no overlapping patients. All patients in the IHC cohort were diagnosed with primary operable breast cancer between 1980 and 1999 and received standard adjuvant treatment according to protocols at the time of diagnosis. Only patients with full clinical data available were included in analysis. All tissue samples were taken at the time of surgical resection, assessed and determined by a pathologist.

### Quantitative reverse transcriptase PCR

#### Tissue processing

After resection of the primary tumour, representative parts of malignant and non-malignant breast tissue were identified by a pathologist, snap frozen and stored in liquid nitrogen. Normal breast tissue was selected and taken from different sites of breast reduction specimens.

#### RNA isolation

Total mRNA was extracted from 50 to 75 mg of breast tissue using the TRIZOL (Invitrogen, Paisley, UK) method according to manufacturer's protocol. RNA quantity and quality was assessed by UV spectrometry (GeneQuant machine, GE Healthcare, Little Chalfont, UK) and by examination of rRNA bands after agarose gel electrophoresis. Only samples that showed both 18S band and a stronger expressed 28S band were used.

#### cDNA synthesis

A measure of 1000 ng of RNA was treated with RNAse-free DNAse and removal reagent kit (Applera, Warrington, UK), and random hexamer primers (50 ng) were used for First Strand cDNA synthesis using SuperScript II RT (Invitrogen). Before using cDNA for PCR amplification, 2 U of RNase H (Applera) were added to samples and incubated for 20 min at 37°C. Quality of cDNA was assessed by using a PCR control run with human *β*-actin. Product bands were assessed by examination of agarose gel electrophoresis. Only samples that showed equal product bands at 330 bp were included in this study.

#### Quantification of mRNA

Real-time quantitative PCR was performed using an ABI Prism 7900 Sequence Detection System (Applied Biosystems, Warrington, UK) and TaqMan Gene Expression Assays (Applied Biosystems) (Table 1). For TaqMan Gene Expression assays, the manufacturer's protocol with recommended 40 rounds of amplification was used. Thermal cycler conditions were 50°C for 2 min, 95°C for 10 min, followed by 40 × 95°C for 15 s and 60°C for 1 min. Product melting curve analysis and gel electrophoresis experiments were used to ensure that only one product of the expected size was amplified.

Negative controls for each primer were included in each run. Quantitative values were obtained from the threshold cycle (*C*t value) at which an increase in TaqMan probe fluorescent signal was associated with an exponential increase of each individual PCR product reaching a fixed threshold value. Each individual primer had a fixed threshold *C*t value. These fixed threshold values were used for every cDNA sample (Table 1).

To enable comparison of different mRNA expression levels, their relation to the average expression level of housekeeping gene *HPRT* (hypoxanthine–guanine phosphoribosyltransferase) was evaluated. Data were analysed using the sequence detection software, which calculates the *C*t value. The expression of the target assay was normalised by subtracting the *C*t value of the housekeeping gene from the *C*t value of the relevant target assay. The fold increase, relative to the control, was obtained by using the formula 2^−Δ*C*t^, and then expressed as a percentage ( × 100). All samples were measured in duplicates.

### Tissue microarray construction

Tissue microarrays (TMAs) were already available for use in this study. The pathologist identified 0.6 mm^2^ cores of breast cancer tissue. The TMA blocks were constructed in triplicates (Tovey *et al*, 2005).

### Immunohistochemistry

Staining for ER, PgR and HER2 had been previously performed for the cohort (Tovey *et al*, 2005). SFK member expression was investigated using antibodies for c-Src (36D10, Cell Signalling Technology, Danvers, MA, USA), Lyn (BD Biosciences, Oxford, UK) and Lck (Cell Signalling Technology). Ki67 antigen MIB-1 (DAKO, Ely, UK) was used to determine proliferation status. All SFK antibodies used were tested by western blot to ensure that only one single band of the appropriate size was observed. Tissue sections were dewaxed and rehydrated through graded alcohol. c-Src antibody was incubated in a preheated antigen retrieval solution (citrate buffer, pH 6.0, Vector Laboratories, Burlingame, CA, USA), whereas Lyn, Lck and Ki67 antibodies were incubated in TE Buffer (pH 8.0, 5 mM Tris, VWR, Lutterworth, UK and 1 mM EDTA, Sigma, Dorset, UK). Antigen retrieval was performed by heating tissue sections under pressure for 5 min in a microwave. Endogenous peroxidase activity was destroyed by incubation in 0.3% hydrogen peroxide (H_2_O_2_) (c-Src), 1% H_2_O_2_ solution (Lyn) and 3% H_2_O_2_ solution (Lck, Ki67), and non-specific binding blocked by incubating in 1.5% normal horse serum (c-Src) and 5% normal horse serum (Lyn, Lck and Ki67) (Vector Laboratories) for 20 min at room temperature. Primary antibody was applied and tissue incubated with c-Src (1 : 200 dilution, 4.32 *μ*g ml^−1^), Lck (1 : 50, 0.76 *μ*g ml^−1^) and Ki67 (1 : 150, 533.33 *μ*g ml^−1^) for 60 min at room temperature. Lyn was incubated overnight at 4°C (1 : 5, 50 *μ*g ml^−1^). Signal was amplified and visualised using the DAKO Envision Kit (DAKO Cytomation, Glostrip, Denmark) and the chromagen 3,3′-diaminobenzidine (DAB, Vector Laboratories). Sections were counterstained, dehydrated and mounted. In each run, a positive control and a negative isotype-matched control was included to ensure no false-positive staining.

### Scoring

The SFK member expression of each core (three per tumour specimen) was assessed using the weighted histoscore method (*H*-score method (Kirkegaard *et al*, 2006)). Ki67 was scored counting positive and negative nuclei in the tumour specimen and then the percentage of positive cells was calculated (Ki67 labelling index (Canna *et al*, 2008)). Agreement between observers was excellent and measured in interclass correlation coefficient (ICCC). All ICCC scores were above 0.8. The observers were blinded to the clinical outcome of the patients.

### Statistical analysis

Differences in expression levels were analysed using the Mann–Whitney *U*-test or Kruskal–Wallis test, including a Wilcoxon-type test for trends, when appropriate. Associations between continuous variables were assessed with the Spearman rank test. Disease-specific survival rates were generated using the Kaplan–Meier method. The log-rank test was used to compare significant differences between subgroups using univariate analysis. Multivariate stepwise Cox-regression analysis was performed to identify factors that were independently associated with disease-specific death. A stepwise backward procedure was used to derive a final model of the variables that had a significant independent relationship with survival. To remove a variable from the model, the corresponding *P*-value had to be >0.05.

Inter-relationships between clinical parameters, ER, PgR and HER2 status were calculated using the *χ*^2^-test. Because of the number of statistical comparisons, a *P*-value of <0.01 was considered to be significant. Data are expressed as median and range. The statistical analyses were performed using a statistical software package (SPSS 15.0 Inc, Chicago, IL, USA).

## Results

### Clinicopathological details of cohort one

The PCR cohort consisted of 81 invasive breast cancers (M), 48 non-malignant (NM) and 10 normal (N) breast tissue samples. Median age of the breast cancer patients was 61 years (IQR 49–74). Median size of breast cancer was 30 mm (IQR 20–42). In all, 40% of the specimens were pathologically graded G2 and 48% G3. A total of 52 breast cancer patients were ER positive compared with 29 ER-negative patients. A total of 55% of breast cancer patients were axillary lymph node positive. Median NPI was 4.6 (IQR 4.3–5.4). Patients underwent either breast-conserving wide local excision (16%) or a radical mastectomy (67% the rest 17% unknown). Axillary dissection was performed in 83% of cases. At time of analysis, 37 out of 79 patients were deceased. Of those 37 patients, 18 died of breast cancer-related causes. Median follow-up time was 5.6 years (IQR 1.8–17.6).

Median age of breast cancer patients, from whom a non-malignant specimen of breast tissue was obtained, was also 61 years (IQR 52–71). Of those patients, 63% were ER positive and 17% ER negative. ER status was not significantly different between tissue types (*P*=0.847). Median age of breast reduction patients supplying normal breast tissue was 37 years (IQR 33–48).

### mRNA expression levels in human breast tissue

**Figure 1:**
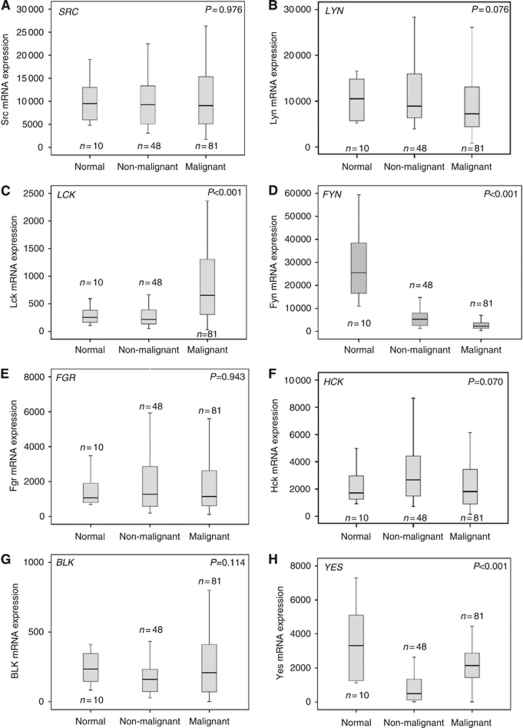
Graphs for cohort one (mRNA). (**A**–**H**) mRNA expression of Src kinase family members in different breast tissue. (**A**) Unchanged *SRC* mRNA expression levels in normal, non-malignant and malignant breast specimens (*P*=0.907). (**B**) *LYN* mRNA expression levels in normal, non-malignant and malignant breast specimens (*P*=0.076). (**C**) Different *LCK* mRNA expression in normal, non-malignant and malignant breast specimens (*P*<0.001). (**D**) Altered *FYN* mRNA expression levels in normal, non-malignant and malignant breast specimens (*P*<0.001). (**E**) *FGR* mRNA expression levels in normal, non-malignant and malignant breast specimens (*P*=0.943). (**F**) *HCK* mRNA expression levels in normal, non-malignant and malignant breast specimens (*P*=0.070). (**G**) *BLK* mRNA expression levels in normal, non-malignant and malignant breast specimens (*P*=0.114). (**H**) *YES* mRNA expression in normal, non-malignant and malignant breast specimens (*P*<0.001).

Expression levels for SFK member were quantified in all tissue samples (Table 1). *BLK* was the least-expressed SFK member in all breast tissues. No change in the level of *SRC* expression was observed between tissue types (*P*=0.976) (Figure 1A), whereas *LCK*, *FYN* and *YES* showed significant changes in expression between different breast tissue types (Table 1). Figure 1A–H demonstrates the data range of all SFK members.

#### SFK member expression in breast cancer specimens

**Figure 2:**
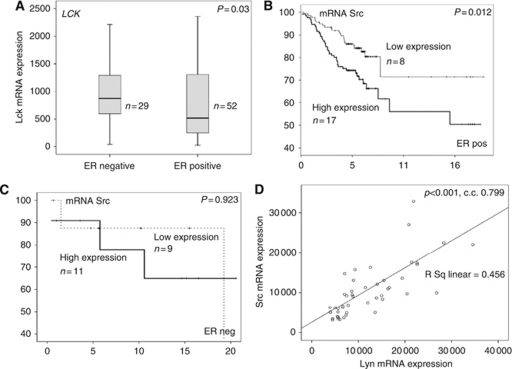
(**A**) The difference between *LCK* mRNA expression in ER-negative compared with ER-positive breast cancer patients (*P*=0.030). (**B**) A Kaplan–Meier survival graph for mRNA expression of SFK member *SRC* in ER-positive patients (*P*=0.012). (**C**) A Kaplan–Meier survival graph for mRNA expression of SFK member *SRC* in ER-negative patients (*P*=0.923). (**D**) The correlation of *SRC* mRNA expression with *LYN* in the non-malignant PCR cohort (*P*<0.001, c.c. 0.799).

The most highly expressed SFK members in malignant breast tissue were *SRC* and *LYN*. Higher expression levels of *LCK* were observed in invasive breast cancers compared with non-malignant and normal breast tissue (*P*<0.001) (Figure 1C). Interestingly, *LCK* was 14-fold less expressed than *SRC*. It also was the only SFK member that showed a difference in expression levels between ER-negative and ER-positive patients. *LCK* was more highly expressed in ER-negative patients, compared with ER-positive patients (Figure 2A). All SFK members correlated with *SRC* expression. The strongest correlation with *SRC* expression was detected with *LYN* (*P*<0.001, c.c. 0.570) and the weakest with *YES* (*P*=0.030, c.c. 0.242). Survival analysis was completed for all SFK members. Only *SRC* was significantly associated with decreased disease-specific survival in ER-positive breast cancer patients (*P*=0.012; Figure 2B) compared with ER-negative patients (*P*=0.923, Figure 2C). Patients with high *SRC* mRNA expression had a median survival of 4.5 years (IQR 2.7–6.3) compared with those with low expression, with a median survival of 11.6 years (IQR 6.9–13.3) (*P*=0.012).

#### SFK member expression in non-malignant breast tissue

As observed within the invasive breast cancer specimen, *SRC* and *LYN* were the highest-expressed SFK members in non-malignant breast tissue. *YES* was least expressed in non-malignant breast tissue, compared with malignant and normal breast tissue (*P*<0.001) (Figure 1H). As with the invasive breast cancer specimens, all other SFK members correlated with *SRC* expression. Again the strongest *SRC* expression correlation was with *LYN* (*P*<0.001, c.c. 0.799) (Figure 2D) and the weakest with *YES* (*P*=0.027, c.c. 0.326).

#### SFK member expression in normal/breast reduction tissue

*FYN* was the most highly expressed SFK member in normal tissue. It was significantly higher expressed than any other SFK members: 2.7-fold higher than *SRC* and 100-fold higher than *LCK*. Highest expression levels of *FYN* were observed in normal tissue compared with non-malignant and lowest in invasive breast cancer specimens (*P*<0.001) (Figure 1D). No correlations between *SRC* and SFK members were observed in normal breast tissue.

### Clinicopathological details of cohort two

The second cohort consisted of 274 breast cancer patients (180 ER positive and 94 ER negative) (Table 2). Median age was 58 years (IQR 51–68). Median tumour size was 20 mm (IQR 15–30). Majority of the cancer specimens were pathologically graded as G2 (45%) and G3 (48%). A total of 49% of the patients were axillary lymph node positive. Mean patient follow-up was 6.3 years (minimum follow-up was 3.7 years and the maximum follow-up was 19.5 years). A total of 17 patients were lost to follow-up. During this period, 65 patients died of their cancer and a further 27 patients died of inter-current disease. In all, 171 patients were still alive at time of last follow-up. Correlations between the clinicopathological characteristics of this cohort are shown in Table 2.

### Protein expression levels of SFK members in invasive breast cancers

#### c-Src kinase

**Figure 3:**
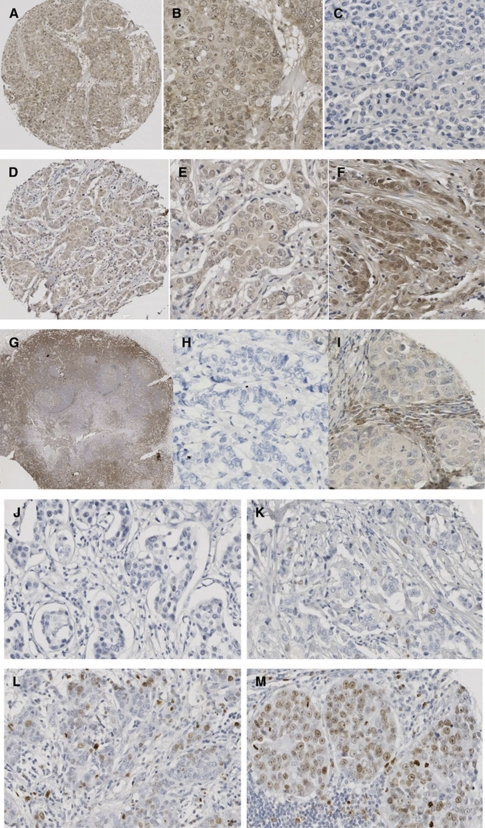
Images of immunohistochemistry (IHC) for each antibody. (**A**–**C**) Breast cancer tissue stained with c-Src antibody (1 : 200, Cell Signalling). (**A**) An overview of a 0.6 mm core of the breast cancer tissue microarray, demonstrating no stromal staining, weak cytoplasmic, none and weak nuclear staining; magnification × 10. (**B**) Weak cytoplasmic, none and weak nuclear and weak membrane staining; magnification × 100. (**C**) Negative staining of stroma and tumour tissue; magnification × 100. (**D**–**F**) Breast cancer tissue stained with Lyn antibody (1 : 5, BD Biosciences). (**D**) An overview of a 0.6 mm core of the breast cancer tissue microarray, demonstrating no stromal staining, weak cytoplasmic, none and weak nuclear staining; magnification × 10. (**E**) Weak cytoplasmic, none and weak nuclear staining; magnification × 100. (**F**) No stromal staining, weak cytoplasmic, none, weak and moderate nuclear staining; magnification × 100. (**G**–**I**) Breast cancer tissue stained with Lck antibody (1 : 50, Cell Signalling). (**G**) Strong staining of tonsil with Lck (positive control); magnification × 2. (**H**) Negative staining of stroma and tumour tissue; magnification × 100. (**I**) Weak cytoplasmic and weak membrane staining; magnification × 100. (**J**–**M**) Ki67 staining of invasive breast cancer specimen (1 : 150, DAKO). (**J**) Negative staining of stroma and tumour tissue; magnification × 100. (**K**) Ki67 staining classified as weak staining; magnification × 100. (**L**) Ki67 staining classified as moderate staining; magnification × 100. (**M**) Ki67 staining classified as strong staining; magnification × 100.

**Figure 4:**
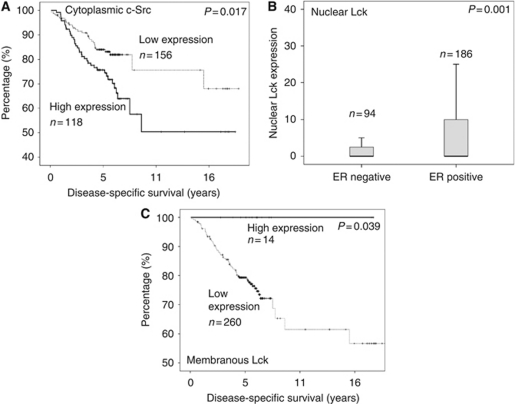
Kaplan–Meier survival graphs for Src kinase family member expressions in cohort two/IHC cohort (*n*=274). (**A**) Kaplan–Meier survival graph for cytoplasmic c-Src (*P*=0.013). (**B**) Boxplot displaying nuclear Lck protein expression difference in ER-negative and -positive breast cancer patients (*P*=0.001). (**C**) Kaplan–Meier survival graph of membranous Lck (*P*=0.039).

Each cellular location was independently assessed for Src kinase expression levels. A total of 48% of tumours exhibited nuclear expression, 61% cytoplasmic and 41% membrane (Figure 3A–C). Tumours were subdivided into those with high (above the median) or low (below or equal to the median) expression. The *χ*^2^-analysis demonstrated that grade and HER2 status positively correlated with cytoplasmic c-Src expression (Table 3). The ER and PgR status correlated negatively with cytoplasmic and membrane c-Src expression (Table 3). c-Src expression at each cellular location correlated with HER2 status (Table 3) and with each other (Table 4). On univariate analysis, neither membrane nor nuclear c-Src expression was associated with disease-specific survival. High cytoplasmic c-Src kinase expression was significantly associated with shorter disease-specific survival (*P*=0.013; Figure 4A), but was not independent in multivariate analysis (Table 2). Those patients with high cytoplasmic c-Src expression had a median survival of 12.2 years (IQR 10.0–14.4) compared with those with low expression, with median survival of 15.6 years (IQR 13.9–17.3).

#### Lyn

Owing to tissue limitations, only 68% of the tumours previously available for analysis were able to be stained for Lyn expression (186 of 274). A total of 34% of Lyn expression was observed in the nucleus, 36% in the cytoplasm and only 5% in the membrane (Figure 3D–F). On univariate analysis, there was no association noticed between Lyn expression and disease-specific survival at any cellular location (Table 2). The *χ*^2^-analysis showed no significant correlations between Lyn expression, clinicopathological features of the cohort or expression level and location of other SFK members (Tables 3 and 4).

#### Lck

Lck expression was observed to be 42.5% in the breast cancers nucleus, 42.5% in the cytoplasm and 5% in the membrane (Figure 3G–I). The *χ*^2^-analysis showed that nuclear Lck expression correlated negatively with ER status (*P*=0.001; Table 3, Figure 4B). Lck expression at all cellular locations correlated with each other. On univariate analysis, cytoplasmic and nuclear Lck expression was not associated with disease-specific survival (Table 2), whereas membrane Lck expression was significantly associated with disease-specific survival (*P*=0.039; Figure 4C). None of patients expressing Lck on the cell membrane died of breast cancer. Median follow-up for patients with positive Lck staining of the membrane was 7.8 years (s.d.±4.5) and for patients without staining, 6.0 years (s.d.±3.5).

#### Ki67

As observed before with Lyn, Ki67 immunohistochemistry was obtained from 71% (194 of 274) of the TMA tumours because of tissue limitation. Median Ki67 score was 3.8 (IQR 0–9.8). Scores were classified into three groups (low-medium-high). A total of 59% of the tumour specimens had a low proliferation rate, with a Ki67 score of <5, 32% had a medium proliferation rate of 5–20 and only 9% of the tumours had a high proliferation rate of >20 (Figure 3J–M). The *χ*^2^-analysis demonstrated that Ki67 scores correlated positively with tumour grade (Table 3) and nuclear c-Src (*P*=0.001). On univariate analysis, Ki67 score was not associated with disease-specific survival.

**Figure 5:**
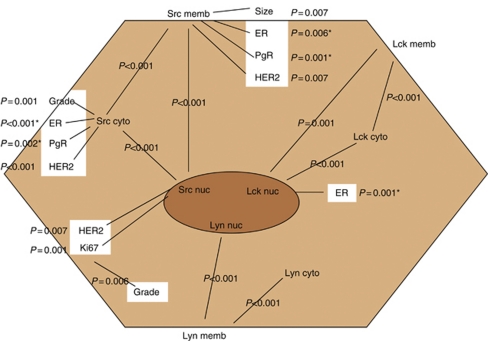
Schematic representation of correlations between clinicopathological features of the IHC cohort, SFK protein expression and cellular location.

The relationship between all family members and clinical parameters is shown in Figure 5.

## Discussion

The SFK members are expressed in various cell types and tissues (Irby and Yeatman, 2000) and involved in cancer progression, via transduction of signals for cell growth, differentiation and survival, influencing cellular adhesion, migration and invasion (Brown and Cooper, 1996). However, there is little translational evidence of SFK member expression in breast tissue. We have investigated expression levels for eight SFK members in normal, non-malignant and malignant breast tissue. Interestingly, *SRC* expression levels were unchanged between the tissue types, despite being the highest-expressed SFK member in malignant and non-malignant, but not in normal breast tissue. *SRC* also was the only SFK member that was significantly associated with patients’ survival. Owing to small patient numbers in the PCR cohort, the study to investigate the role of Src was expanded into a larger cohort of formalin-fixed paraffin-embedded specimens (IHC cohort). High expression of cytoplasmic c-Src was associated with shorter disease-specific survival, increasing grade, tumour size, ER negativity and HER2 positivity. These findings are consistent with our previous results (Elsberger *et al*, 2009), with c-Src being associated with more aggressive growth in cancer cell lines (Hynes, 2000; Frame, 2002). We observed that Ki67 proliferation index correlated positively with c-Src nuclear expression, but not with c-Src cytoplasmic expression. This suggests that c-Src may have multiple roles within the cell depending on cellular location. It was not surprising to find that Ki67 was positively correlating with grade of the breast cancers.

*LYN* was also expressed at high mRNA levels in M and NM tissue. However, no associations with clinicopathological features and survival were noted in both cohorts. Other studies report that Lyn has a part in developing chemoresistance of colon cancer (Bates *et al*, 2001), progression of prostate tumours (Park *et al*, 2008) and leukaemia (Lee *et al*, 2008). The latter is not surprising, as Lyn is associated with a number of haematopoietic cell surface receptors, cytokine receptors and is a key mediator in several pathways of B-cell activation (Hibbs and Dunn, 1997). These observations are congruent with a role for Lyn associated with the development of chemoresistance. This study does not use any hormone-resistant or chemoresistant tumours; only tumours obtained at primary diagnosis. In the RT–PCR cohort, *LCK* mRNA expression in invasive breast cancer was 14-fold less than *SRC* and was associated with improved disease-specific survival in the IHC cohort. Surprisingly, this was reverse to the observation made with c-Src. Lck membrane expression was not associated with patient mortality. A similar immunohistochemistry study (Elsberger *et al*, 2009), investigating the different phosphorylation sites of Src, demonstrated that phosphorylation site Y215 was associated with improved disease-specific survival. With the knowledge that phosphorylation antibodies are able to detect other SFK members, we hypothesise that the Y216Src antibody used could have identified Lck as the other SFK member associated with good clinical outcome. However, this observation with Lck membrane expression and good prognosis was only observed in a small number of patients. Therefore, further analysis in a much larger cohort is required to verify these results.

*LCK* mRNA expression was found to be higher in ER-negative compared with ER-positive breast cancer samples. This was not reproducible in the IHC cohort. Nuclear Lck protein expression was higher expressed in the ER-positive breast cancer patients. Interestingly, survival analysis with high nuclear Lck expression displayed a trend to better clinical outcome of ER-positive compared with ER-negative patients within the first 5 years after diagnosis (data not shown). These findings are consistent with results reported by Rody *et al* (2009), showing that Lck was associated with improved disease-free survival within the ER-positive and ER/HER2-positive breast cancer subgroup. It is unclear whether this discrepancy between mRNA and protein expression is based on using different patient cohorts or altered transcription from RNA level to protein synthesis and expression.

As Lck is known to be involved in T-cell proliferation (Palacios and Weiss, 2004), it was hypothesised that expression of this gene was linked with the presence of lymphocyte infiltration within the tumour and interacting with it. Results of this study demonstrated that Lck is expressed within the tumour, providing evidence that Lck itself may be involved with signal transduction in the tumour. Other studies suggest that Syk, a member of another non-receptor tyrosine family, is involved in hypoxia-driven tumour progression via cross-talk to Lck in the nucleus (Chakraborty *et al*, 2006).

*Lck* has been implicated in the mitochondrial apoptosis pathway by controlling the expression of pro-apoptotic factor Bak (Samraj *et al*, 2006). Cell line experiments have shown that *Lck* deficiency resulted in resistance to anticancer drug-induced apoptosis. T-lymphoma cells lacking Lck have shown marked resistance to apoptosis reduction on exposure to ionising radiation (Belka *et al*, 1999). A more recent study from the same group adjusted their previous findings that not just the lack of Lck caused pronounced apoptosis resistance in response to stimuli of the intrinsic pathway but the additional loss of Bak was responsible for reduced sensitivity (Rudner *et al*, 2009). Decreased levels of other pro-apoptotic Bcl-2 family members, for example, Bax, have been shown to correspond to shorter survival in women with metastatic breast cancer. However, as yet no significant correlation between Bak and clinical outcome has been seen within breast cancer (Reed, 1999).

This study established that all SFK members were expressed in normal, non-malignant and malignant breast tissue. Quantitative real-time PCR results showed equal *SRC* mRNA expression within the different breast tissue types. However, this method does not provide details regarding cellular location of Src kinase. Despite two dissimilar cohorts of breast cancer patients and different investigation methods, Src kinase was associated with poorer survival outcome.

One major problem still remains: how to assess which tumours will respond to Src inhibitors, so that those patients can be selected who will probably benefit most from the treatment. Applicability of microarray gene analysis in the clinical setting is now being tested after identifying a gene signature that was able to predict response to dasatinib in cell lines (Huang *et al*, 2007). Response rate with molecular target therapy in unselected patient groups have been very modest so far, and this most likely indicates that in most solid tumours no single molecular event drives tumourigensis. The need for new predictive and prognostic indicators is clear, especially in the currently difficult-to-treat patient subgroups such as ER/PgR-negative and/or HER2-positive patients. The results of this study highlight an exciting area for future research in terms of predicting survival and the mechanisms of how this occurs.

## Figures and Tables

**Table 1:** Intron-skipping primers used for real-time PCR, their fixed threshold *C*t values and expression levels in different breast tissue

**Gene**	**Gene expression assay ID**	**Threshold (*C*t) value**	**Expression levels in M**	**Expression levels in NM**	**Expression levels in N**	***P*-values**
*SRC*	Hs00178494_m1	0.28263707	9041	9252	9493	0.976
*LYN*	Hs00176719_m1	0.34538345	7233	8922	10 521	0.076
*LCK*	Hs00178427_m1	0.26297827	655	217	255	**<0.001**
*FYN*	Hs00176628_m1	0.27740355	2245	5293	25 484	**<0.001**
*FGR*	Hs00178340_m1	0.22993330	1144	1275	1072	0.943
*HCK*	Hs00176654_m1	0.37934458	1815	2673	1712	0.070
*BLK*	Hs00176441_m1	0.23531063	208	161	234	0.114
*YES*	Hs00736972_m1	0.14854589	2142	499	3312	**<0.001**
*HPRT*	4310890E	0.25742040	NA	NA	NA	NA

Abbreviations: M=invasive breast cancer; N=normal tissue; NA=not applicable; NM=non-malignant.

The table reveals details of each individual Src kinase family member and the housekeeping gene *HPRT*. Expression levels of Src kinase family members in different breast tissue types are stated as medians. *P*-values express alterations of expression in the different breast tissue types (Kruskal–Wallis test). Bold typeface is used to highlight significant *P*-values.

**Table 2:** Impact of clinicopathological factors and protein expression/activation on patient survival

**Total patient cohort (274 patients)**		**Univariate analysis**			**Multivariate analysis**		
**Variables**	**Numbers**	***P*-value**	**HR**	**IQR**	***P*-value**	**HR**	**IQR**
Age (<50/>50 years)	68/206	0.365	1.3	0.7–2.4			
Tumour type (ductal/lobular/tubular/others)	260/9/2/3	0.774	0.7	0.3–1.6			
Grade (G1/G2/G3)	20/122/132	**0.030**	1.8	1.2–2.8	0.659		
Size (<20/20–50/>50 mm)	111/148/15	**<0.001**	3.8	2.4–6.0	**<0.001**	12.2	5.4–27.8
Lymph node (positive/negative)	132/142	**<0.001**	2.5	1.5–4.1	**0.018**	1.9	1.1–3.2
ER status (positive/negative)	180/94	**<0.001**	2.3	1.4–3.8	**<0.001**	2.5	1.5–4.1
PgR status (positive/negative)	132/140	**0.009**	1.9	1.2–3.2	0.579		
HER2 status (positive/negative)	38/236	**0.001**	2.5	1.4–4.3	0.281		
c-Src nuc expression (positive/negative)	129/145	0.399	1.2	0.7–2.0			
c-Src cyto expression (positive/negative)	118/156	**0.013**	1.8	1.1–3.0	0.131		
c-Src memb expression (positive/negative)	113/161	0.787	1.9	1.2–3.2			
Lyn nuc expression (positive/negative)	66/116	0.742	0.9	0.5–1.7			
Lyn cyto expression (positive/negative)	61/121	0.997	1.0	0.5–1.9			
Lyn memb expression (positive/negative)	9/173	0.825	1.1	0.3–3.7			
Lck nuc expression (positive/negative)	116/158	0.148	0.7	0.4–1.1			
Lck cyto expression (positive/negative)	117/157	0.964	1.0	0.9–1.0			
Lck memb expression (positive/negative)	14/260	**0.039**	0.1	0.01–4.0	0.972		
Ki67 (<5/5–20/>20)	261/91/30	0.254	1.0	0.5–1.9			

Abbreviations: c-Src=total Src kinase; c-Src cyto=c-Src cytoplasmic expression; c-Src memb=c-Src membrane expression; c-Src nuc=c-Src nuclear expression; ductal=ductal carcinoma; ER=oestrogen receptor; Grade=Bloom and Richardson grade; HER2=human epidermal growth factor receptor 2; HR=hazards ratio; IQR=interquartile range; Ki67=Ki67 proliferation score, low <5, moderate 5–20, high >20; Lck cyto=Lck cytoplasmic expression; Lck memb=Lck membrane expression; Lck nuc=Lyn nuclear expression; Lyn cyto=Lyn cytoplasmic expression; Lyn memb=Lyn membrane expression; Lyn nuc=Lyn nuclear expression; others=mucinous, mucoid and micropapillary carcinoma; PgR=progesterone receptor; tubular=tubular carcinoma.

The table shows an overview of the full patient cohort's characteristics. Each clinical and pathological parameter was correlated to disease-specific survival (*P*-values). Bold typeface is used to highlight significant *P*-values.

**Table 3:** The interrelationships between the clinicopathological characteristics of patients with breast cancer, Src kinase family member expression and Ki67

**Total patient cohort (274 patients)**		***χ*^2^ *P*-values**									
**Variables**	**Numbers**	**c-Src nuc**	**c-Src cyto**	**c-Src memb**	**Lyn nuc**	**Lyn cyto**	**Lyn memb**	**Lck nuc**	**Lck cyto**	**Lck memb**	**Ki 67**
Age (<50/>50 years)	68/206	0.786	0.638	0.798	0.259	0.027	0.297	0.639	0.927	0.024	0.965
Tumour type (duct/lob/tub/others)	260/9/2/3	0.788	0.431	0.446	0.642	0.547	0.492	0.375	0.032	0.842	0.360
Grade (G1/G2/G3)	20/122/132	0.021	**0.001**	0.017	0.019	0.632	0.991	0.034	0.543	0.741	**0.006**
Size (<20, 20–50, >50 mm)	111/148/15	0.421	0.037	**0.007**	0.029	0.832	0.115	0.892	0.580	0.926	0.186
Lymph node (positive/negative)	132/142	0.732	0.359	0.589	0.123	0.200	0.247	0.957	0.308	0.311	0.590
ER status (positive/negative)	180/94	0.043	**<0.001** a	**0.006** a	0.133	0.122	0.876	**0.001** a	0.600	0.118	0.997
PgR status (positive/negative)	132/140	0.184	**0.002** a	**0.001** a	0.339	0.015	0.048	0.097	0.036	0.090	0.280
HER2 status (positive/negative)	38/236	**0.007**	**<0.001**	**0.007**	0.733	0.836	0.772	0.168	0.085	1.000	0.200

Abbreviations: c-Src=total Src kinase; c-Src cyto=c-Src cytoplasmic expression; c-Src memb=c-Src membrane expression; c-Src nuc=c-Src nuclear expression; duct=ductal carcinoma; ER=oestrogen receptor; Grade=Bloom and Richardson grade; HER2=human epidermal growth factor receptor 2; Ki67=Ki67 proliferation score, low <5, moderate 5–20, high >20; Lck cyto=Lck cytoplasmic expression; Lck memb=Lck membrane expression; Lck nuc=Lyn nuclear expression; lob=lobular carcinoma; Lyn cyto=Lyn cytoplasmic expression; Lyn memb=Lyn membrane expression; Lyn nuc=Lyn nuclear expression; others=mucinous, mucoid and micropapillary carcinoma; tub=tubular carcinoma.

a Negative correlation.

Bold typeface is used to highlight significant *P*-values.

**Table 4:** The interrelationships between Src kinase family member expression and Ki67

**Total patient cohort (274 patients)**		***χ*^2^ *P*-values**								
**Variables**	**Numbers**	**c-Src cyto**	**c-Src memb**	**Lyn nuc**	**Lyn cyto**	**Lyn memb**	**Lck nuc**	**Lck cyto**	**Lck memb**	**Ki67**
c-Src nuc (positive/negative)	129/145	**<0.001**	**<0.001**	0.163	0.646	0.917	0.328	0.461	0.701	**0.001**
c-Src cyto (positive/negative)	118/156		**<0.001**	0.621	0.579	0.780	0.299	0.508	0.934	0.110
c-Src memb (positive/negative)	113/161			0.691	0.497	0.410	0.274	0.064	0.017	0.110
Lyn nuclear (positive/negative)	66/116				**<0.001**	0.345	0.016	0.975	0.534	0.081
Lyn cyto (positive/negative)	61/121					**<0.001**	0.424	0.067	0.032	0.598
Lyn memb (positive/negative)	9/173						0.282	0.726	0.053	0.010
Lck nuclear (positive/negative)	116/158							**<0.001**	**0.001**	0.045
Lck cyto (positive/negative)	117/157								**<0.001**	0.572
Lck memb (positive/negative)	14/260									0.506

Abbreviations: c-Src=total Src kinase; c-Src cyto=c-Src cytoplasmic expression; c-Src memb=c-Src membrane expression; duct=ductal carcinoma; c-Src nuc=c-Src nuclear expression; Grade=Bloom and Richardson grade; Ki67=Ki67 proliferation score low<5, moderate 5–20, high >20; Lck cyto=Lck cytoplasmic expression; Lck memb=Lck membrane expression; Lck nuc=Lyn nuclear expression; lob=lobular carcinoma; tub=tubular carcinoma; Lyn cyto=Lyn cytoplasmic expression; Lyn memb=Lyn membrane expression; Lyn nuc=Lyn nuclear expression; others=mucinous, mucoid and micropapillary carcinoma.

Bold typeface is used to highlight significant *P*-value.
